# An Unconventional Solution for Persistent Lateral Hip Prosthetic Friction Syndrome (LHPFS) after Revision Total Hip Arthroplasty

**DOI:** 10.1155/2024/7934419

**Published:** 2024-04-18

**Authors:** Matthias Wittauer, Karl Stoffel

**Affiliations:** ^1^Department of Trauma and Orthopaedic Surgery, University Hospital Basel, Basel, Switzerland; ^2^University of Basel, Basel, Switzerland

## Abstract

We report on a 77-year-old male patient, who presented with excessive bone loss at the area of the greater trochanter after several hip revision surgeries resulting in a persistent friction syndrome caused directly by the rough surface and sharp edges of the prosthetic shoulder of a well-fixed Wagner-type revision stem. Surgery was performed by creating a cemented neotrochanter with an attached polyester patch around the proximal lateral shaft and performing a Z-plasty of the iliotibial tract. Twelve months postoperatively, the patient reported a reduction in subjective pain of 50% and improvement of the Harris Hip Score from 45 to 75 points. Without a definition in the current literature, the authors propose the term “lateral hip prosthetic friction syndrome” (LHPFS) to describe this medical condition.

## 1. Introduction

Persistent pain around the greater trochanter area after total hip arthroplasty is a fairly common complication, with an incidence of 4-17% and a significant effect on mobility and quality of life [1–3]. In recent years, the former term of “trochanteric bursitis” has been substituted by greater trochanteric pain syndrome (GTPS). Its etiology is not fully understood and can be multifactorial. Greater trochanteric pain syndrome was for a long time mistakenly attributed to bursal inflammation as its cause. However, recent research has shown that bursal inflammation does not have an etiologic role in this condition [4, 5]. In contrast, friction between the greater trochanter and the overlying iliotibial band is considered a possible cause [6].

Multiple revisions, massive osteolysis, or stress shielding can lead to extensive proximal femoral bone loss, creating a major challenge for revision hip arthroplasty [7, 8]. In patients with proximal femoral bone stock deficiency, long taper-fluted diaphyseally fixed uncemented stems, like the Wagner-type revision stems, provide a good solution to achieve mechanical stability [9, 10]. In those patients, friction between the femoral shoulder of the stem and the overlying soft tissue can cause symptoms similar to GTPS, despite the loss of the greater trochanter.

Being a fairly rare problem in hip revision surgery, it is not yet recognized as a proper entity by the orthopaedic literature and recommendations for operative solutions are scarce.

In this case report, we present a patient whose excessive bone loss at the area of the greater trochanter resulted in a persistent lateral hip prosthetic friction syndrome (LHPFS) caused by the rough surface and sharp edges of the neck-stem junction of a Wagner-type revision stem. The purpose of reporting this case is to offer an unconventional step-wise surgical technique for symptom relief in individuals affected by LHPFS. Simultaneously, our aim is to enhance awareness by introducing a precise medical term for this distinct medical entity, denoted as lateral hip prosthetic friction syndrome.

## 2. Case Presentation

### 2.1. Patient History

A 77-year-old patient was referred to us from a local hospital for treatment of chronic left hip pain after several revision arthroplasties. Ten years earlier, the patient received a primary total hip arthroplasty for left-sided hip osteoarthritis. Revision surgery to address the aseptic loosening of the shaft was undertaken only after a year. During this first revision surgery, the primary shaft was removed, and a Wagner-type shaft (Revitan, Zimmer Biomet, Warsaw, Indiana) was implanted via a transfemoral approach. The osteotomy was secured with cerclage wires. A total of five revision surgeries followed to address various postoperative complications including irritation by cerclage wires, nonunion of the osteotomy, and malrotation of the greater trochanter. Finally, a stable arthroplasty and well-healed femur were achieved at the cost of excessive stress shielding around the greater trochanter.

**Figure 1:**
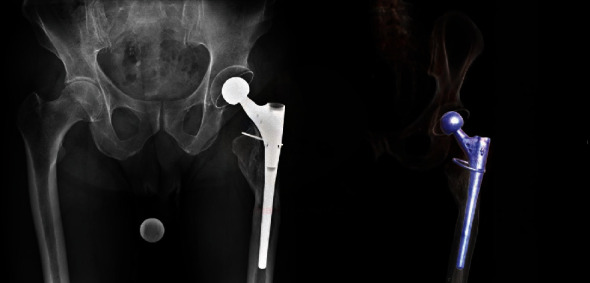
Preoperative AP pelvis X-ray and 3D CT scan showing a well-fixed Wagner-type shaft with excessive bone loss at the area of the greater trochanter (Gruen zones 1 and 2) and diffuse bone remodeling around the medial femoral cortex.

When presenting at our outpatient department, the patient complained about persistent lateral hip pain, reluctant to physiotherapy and oral analgesics. In the physical exam, the patient reported diffuse pain around the iliotibial band, peaking at the palpable part of the proximal shaft component. The patient was assessed using a visual analog scale (VAS) for pain (0–10), and he reported a pain score of 9. The Harris Hip Score amounted to 45 points out of 100. The scar and surrounding soft tissue did not show any signs of inflammation. Clinically, there were no signs of implant loosening. The patient displayed a discrete limp due to pain, yet the Trendelenburg test was negative. A radiographic examination revealed a well-fixed shaft with excessive bone loss at the area of the greater trochanter (Gruen zones 1 and 2) and diffuse bone remodeling around the medial femoral cortex (Figure 1). Hip offset and leg length were well reconstructed. A single cerclage wire to stabilize the former transfemoral osteotomy was still in place.

### 2.2. Preoperative Management

We performed a single photon emission computed tomography (SPECT) and magnetic resonance imaging (MRI), which were able to exclude component loosening and tendon rupture of the gluteal musculature. The MRI did not show evidence of fluid or imbibition of the surrounding fatty tissue, but scarred thickening of the joint capsule and scar plate adjacent to the prosthetic shoulder with signal alteration of the gluteus medius and minimus tendon. Increased fatty atrophy of the left-sided gluteal muscles compared to the opposite side (Goutallier grade II) was noticed as well.

A joint aspiration successfully excluded a periprosthetic joint infection.

After the conservative measurements failed, the patient opted for surgical treatment. The author's experience in previous attempts to treat this specific complication with allograft placement over the prosthetic shoulder was unsuccessful. Prior to surgery, we conducted a literature search using PubMed and found several surgical techniques addressing abductor deficiency in revision hip arthroplasty or treatment options for greater trochanteric pain syndrome [1, 2, 11, 12]. The current literature does not provide any possible treatment strategy for proximal iliotibial band friction syndrome caused directly by a femoral stem. We finally decided to undertake the here-described revision surgery.

### 2.3. Stepwise Surgical Technique

#### 2.3.1. Step 1: Approach

The operation was performed with the patient in a lateral decubitus position. Access to the femoral shaft was obtained via a posterior hip approach (*Kocher-Langenbeck),* through a spindle-shaped incision of the old, retracted scar. Blunt dissection was performed to expose the iliotibial tract, which was split longitudinally. The gluteus maximus was split in line with its fibers, starting at the prosthetic shoulder in a proximal direction. The proximal part of the iliotibial tract appeared to be fused to the hip abductors and was detached manually. Full exposure of the proximal part of the stem was finally achieved. Scarred thickening of the tissue over the prosthetic shoulder was detected as a sign of chronic soft tissue irritation. The cerclage wire was removed, and a moderate degree of local metallosis was noticed.

#### 2.3.2. Step 2: Patch Placement

**Figure 2:**
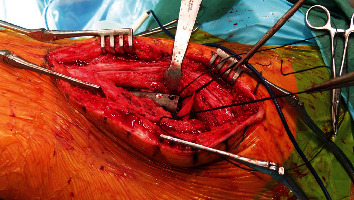
Intraoperative image showing the fixation of a polyester patch to the shaft and joint capsule.

To create a smooth surface allowing soft tissue adherence around the planned neotrochanter, a polyester patch (35 × 25 mm, Pitch-Patch Tissue Reinforcement, Xiros, Leeds, UK) was first fixed medially to provide the possibility to ensheathe the cement circularly. The patch was therefore partly fixed to the shaft with the help of the mounted Ethibond (Ethicon, Somerville, New Jersey) suture and medially fixed to the joint capsule with two sutures, creating a tent-like structure (Figure 2).

#### 2.3.3. Step 3: Creation of a Cemented Neotrochanter

**Figure 3:**
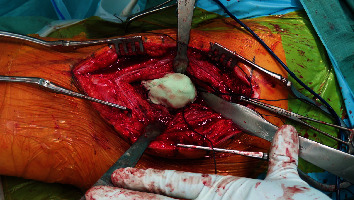
Intraoperative image showing the attachment of a cement block around the proximal lateral shaft creating a neotrochanter.

**Figure 4:**
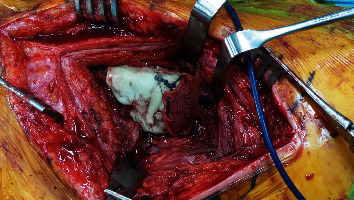
Intraoperative image showing the fixation of the polyester patch on top of the cement block.

Bone cement (PALACOS, Heraeus Medical, UK) was molded around the proximal shaft in the shape of a round capsule, creating a trochanter-like shape (Figure 3). The cement was cautiously inserted into the proximal holes of the stem to create a stable construct. Another Ethibond suture was placed through the cemented neotrochanter, while still being soft. After cement hardening, the polyester patch was then closed over the top by tensioning it to the lateral suture fixed inside the cement. This created a cover over the cement in order to smoothen the surface and to allow adherence of soft tissue (Figure 4). The wound was then extensively irrigated, followed by a side-to-side closure of the scar plate over the lateral vastus/abductors. Finally, a redon drain was inserted, and the iliotibial tract was closed with Vicryl sutures (Ethicon, Somerville, New Jersey).

#### 2.3.4. Step 4: Iliotibial Band Z-Plasty

**Figure 5:**
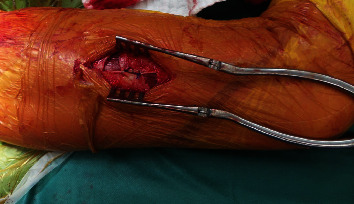
Intraoperative image of the Z-plasty resulting in lengthening of the iliotibial band.

As a next step, a distal lengthening of the iliotibial band was performed doing a Z-plasty as proposed by Sayed-Noor et al. [13]. The ITB margins above the knee joint were palpated, and an 8 cm longitudinal skin incision was performed. After blunt removal of the fatty tissue, a longitudinal incision of about 8 cm was made in the iliotibial band. Another two vertical incisions, the distal one running anteriorly and the proximal one running posteriorly, completed the Z-plasty. This resulted in a lengthening of the iliotibial band of about 2 cm. The longitudinal incision of the iliotibial band was then sutured side to side using a Vicryl suture. The effect of lengthening of the iliotibial band can be seen in the exposure of the underlying musculature as a result of distending the vertical incisions (Figure 5).

Standard skin closure was performed with nonabsorbable sutures. Sterile dressings were applied, and the leg was elastically wrapped.

### 2.4. Aftercare and Follow-Up

Partial weight bearing was allowed with a load of 15 kg for four weeks and a restriction of hip flexion to 70° for six weeks.

**Figure 6:**
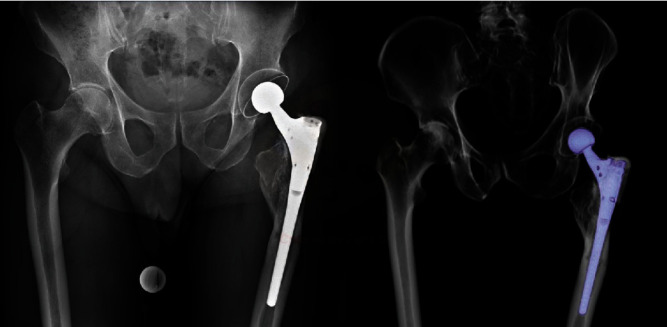
Postoperative AP pelvis X-ray and 3D CT scan showing a well-fixed cemented neotrochanter around the proximal part of the femoral component protecting the sharp edges of the stem.

Postoperative radiographs and CT scans showed a well-fixed cement mantle around the proximal part of the femoral component. The sharp edges were now protected by the rounded cement plug (Figure 6).

Twelve months postoperatively, the patient walked without a limp and reported a reduction in subjective pain of 50%. The initial VAS for pain was reported at 9, and in the follow-up appointment, the subjective pain was reduced to a score of 4. The Harris Hip Score improved from 45 points preoperatively to 75 points postoperatively.

The patient reported overall satisfaction with the surgical result. Informed consent has been obtained from the patient to publish this case report.

## 3. Discussion

Revision hip arthroplasty can pose significant challenges in cases where multiple revisions, massive osteolysis, or stress shielding leads to extensive proximal femoral bone loss. In patients with proximal femoral bone stock deficiency, long taper-fluted diaphyseally fixed uncemented stems, like Wagner-type revision stems, provide a good solution to achieve mechanical stability [9, 10]. In those patients, friction between the femoral shoulder of the stem and the overlying soft tissue can cause symptoms similar to GTPS, despite a loss of the greater trochanter.

Treatment options, outcomes, and risk factors for greater trochanteric pain syndrome after total hip arthroplasty are fairly well described in the orthopaedic literature [1, 2, 14–17]. Worlicek et al. showed that anatomic restauration of leg length and acetabular and femoral offset reduces postoperative trochanteric pain syndrome and improves the clinical outcome of patients [14]. A survey among Canadian arthroplasty surgeons revealed that most commonly, physical examination alone is used for diagnosis [18]. Surface irregularities of the greater trochanter, long believed to be a potential radiographic sign of GTPS, have proven to be an unreliable radiographic indicator for diagnosis [19]. Nevertheless, distension of the greater trochanteric bursa, presenting as a discrete, well-defined, characteristic fluid collection around the greater trochanter, proved to be MR-tomographic evidence of GTPS [20].

Currently, there is no consensus on clinical guidelines for the diagnosis or management of GTPS, and the usual nonsurgical management options for GTPS include physiotherapy, corticosteroid injections, and platelet-rich plasma (PRP) [18, 21]. A recent randomized controlled trial was able to show the superiority of focused shockwave therapy compared to corticosteroid injections in the treatment of GTPS [22]. In general, conservative treatment of lateral trochanteric pain following primary total hip arthroplasty has high success rates [23]. At the same time, surgery for greater trochanteric pain syndrome is related to poor outcomes, significant complications, and concerning reoperation rates [15].

Whereas in GTPS, friction between the greater trochanter and the overlying soft tissue leads to local inflammation and pain, the same soft tissue reaction is possible in patients who lost the greater trochanter, making the prosthesis itself the main source of friction and subsequent pain.

The combination of excessive femoral bone loss in Gruen zones 1 and 2 with a long taper-fluted diaphyseally fixed uncemented stem sometimes can lead to a friction syndrome at the proximal iliotibial band overlying the neck-shaft junction. The current literature does not provide a definition for this medical entity, even though it is a fairly rare, but recurrent, problem in hip revision surgery with excessive bone loss. The authors therefore propose the term “lateral hip prosthetic friction syndrome” (LHPFS) to describe this medical condition.

The introduction of this term addresses a notable gap in the existing literature by offering clinicians standardized terminology to effectively communicate and recognize the condition. With LHPFS defined, clinicians can diagnose the syndrome more accurately, facilitating appropriate management strategies tailored to address its specific characteristics. This not only enhances patient care but also guides treatment protocols, potentially leading to better outcomes for affected individuals. Furthermore, LHPFS fosters patient empowerment by clarifying their condition. With accessible information and a recognized term, patients can actively engage in discussions with healthcare providers, promoting shared decision-making and enhancing overall satisfaction with treatment outcomes.

Before this report, there was no specific term to describe the condition experienced by this patient: persistent lateral hip pain caused directly by friction between the soft tissue and the prosthetic femoral components. By introducing the term LHPFS, the authors provide a concise and descriptive label for this distinct medical entity.

To diagnose LHPFS effectively, it is essential to initially exclude other potential causes of pain after total hip arthroplasty. This necessitates a comprehensive exploration of various underlying factors to precisely identify the pain's origin [24]. A critical aspect of diagnosing LHPFS is the identification of a radiographically absent greater trochanter due to proximal femoral bone loss, which results in an exposed proximal portion of the femoral stem. This unique combination distinguishes LHPFS from GTPS, where the absence of the greater trochanter and irritation caused directly by the prosthesis are key factors. Additionally, during clinical examination, it is imperative to identify localized lateral hip pain specifically over the area where soft tissue directly interfaces with the prosthesis.

In addition to the lack of definition, recommendations for surgical solutions are not available in the current medical literature. In general, surgery should be considered the last option in the treatment of LHPFS. It should only be pursued when conservative measures, such as physiotherapy and steroid injections, have persistently failed to provide relief.

The here-described step-wise surgical technique only requires the placement of a cement block combined with a synthetic patch. Polyester patches are already well established for augmentation in massive rotator cuff tears [25]. Their open-weave design facilitates tissue ingrowth while being well tolerated and devoid of any immunological reactions. By providing a protective barrier between the stem and surrounding tissues, the cemented shield effectively addressed the source of irritation, leading to enhanced comfort and overall patient well-being.

Our proposed surgical technique is easy to perform, not time-consuming, and does not require special orthopaedic devices. Nevertheless, it should be seen as one of several possible surgical solutions for patients suffering from LHPFS. By combining this technique with a distal Z-plasty of the iliotibial band in this patient, confounding must be considered as a possible source of bias. Solely addressing iliotibial band tightness with a Z-plasty could also alleviate symptom severity. However, we contend that eliminating the irritating interaction between the femoral stem and overlying soft tissue remains paramount. While alternative materials, such as tendon or bone allografts, instead of the cement-patch combination for neotrochanter creation, might also show comparable results, they have not yet been described in the literature. Nevertheless, the combination of interventions described in this paper was able to provide a significant reduction in local pain scores and improve patient satisfaction. Further studies, including a series of patients treated with this technique, are needed to assess potential complications and ascertain its efficacy to provide long-term satisfaction in patients suffering from LHPFS.

## 4. Summary

The simple solution of creating a neotrochanter around an irritating metal stem using a combination of cement and a polyester patch was able to significantly reduce local pain scores and improve patient satisfaction in a 77-year-old patient suffering from persistent lateral hip prosthetic friction syndrome (LHPFS). The introduction of LHPFS fills a significant gap in the literature by providing standardized terminology for a previously undefined medical condition. While recognizing the limitations and the need for continued investigation, this case report highlights the clinical relevance of the proposed term and the efficacy of the described surgical technique in managing LHPFS.
